# Zebrafish Müller glia-derived progenitors are multipotent, exhibit proliferative biases and regenerate excess neurons

**DOI:** 10.1038/srep24851

**Published:** 2016-04-20

**Authors:** Curtis Powell, Eli Cornblath, Fairouz Elsaeidi, Jin Wan, Daniel Goldman

**Affiliations:** 1 Molecular and Behavioral Neuroscience Institute, Department of Biological Chemistry, University of Michigan, Ann Arbor, MI 48109 USA

## Abstract

Unlike mammals, zebrafish can regenerate a damaged retina. Key to this regenerative response are Müller glia (MG) that respond to injury by reprogramming and adopting retinal stem cell properties. These reprogrammed MG divide to produce a proliferating population of retinal progenitors that migrate to areas of retinal damage and regenerate lost neurons. Previous studies have suggested that MG-derived progenitors may be biased to produce that are lost with injury. Here we investigated MG multipotency using injury paradigms that target different retinal nuclear layers for cell ablation. Our data indicate that regardless of which nuclear layer was damaged, MG respond by generating multipotent progenitors that migrate to all nuclear layers and differentiate into layer-specific cell types, suggesting that MG-derived progenitors in the injured retina are intrinsically multipotent. However, our analysis of progenitor proliferation reveals a proliferative advantage in nuclear layers where neurons were ablated. This suggests that feedback inhibition from surviving neurons may skew neuronal regeneration towards ablated cell types.

Retinal degenerations are a heterogeneous group of diseases that often lead to blindness. Two of the most common blinding eye degenerations are macular degeneration and glaucoma, which are characterized by photoreceptor and ganglion cell death, respectively. Although a number of strategies are being explored for restoring sight to those suffering from retinal degenerative disease, including prosthetics and stem cell transplants, it seems a regenerative one might be ideal. Unlike mammals, teleost fish, such as zebrafish, can regenerate retinal neurons to replace those lost by injury or disease^1^,^2^. This contrasting outcome results from differences in the injury-response of Müller glia (MG), a radial glia-like cell found in the retinas of both fish and mammals^3^,^4^,^5^,^6^.

In fish, MG respond to retinal injury by undergoing a reprogramming event that is characterized by changes in DNA methylation and gene expression^7^,^8^,^9^,^10^. A variety of signaling cascades, including Notch, MAPK, PI3K, Jak/Stat, and GSK3β/β-catenin, have been identified that help transduce injury signals to the MG genome and initiate a regenerative response^11^,^12^,^13^,^14^,^15^,^16^,^17^. These signaling cascades stimulate MG cell division and the production of retinal progenitors^3^,^18^,^19^. The newly generated MG-derived progenitors undergo a finite number of divisions, a process controlled, at least in part, by Notch, Pax6 and Insm1a, and generate a population of retinal progenitors in the inner nuclear layer (INL)^12^,^20^,^21^. These progenitors migrate to the various retinal nuclear layers, where they differentiate into the retinal layer appropriate neurons^3^,^19^,^22^.

Whether MG-derived progenitors limit their fate to ablated neurons remains unclear. In one study, the selective ablation of UV cones appeared to dramatically skew progenitor fate toward cones^23^. In another study, impaired FGF signaling was reported to cause photoreceptor death and stimulate MG-derived progenitors to selectively migrate to the outer nuclear layer (ONL) and regenerate photoreceptors^24^. And when retinal cell ablation was restricted to neurons residing in the retina’s INL and ganglion cell layer (GCL), excess cells were not observed in the retina’s ONL^22^. These data suggest that progenitors are biased towards regenerating ablated cell types. However, these studies were incomplete in their lineage tracing analysis of progenitors and this may have impacted the interpretation of results.

In contrast, using BrdU lineage tracing strategies to analyze regeneration of all major retinal neuron types, we have reported that growth factor or cytokine treatment of an uninjured retina stimulates MG proliferation and the generation of multipotent progenitors that seed new retinal neurons into all nuclear layers^12^,^14^. These data suggest that MG-derived progenitors may be intrinsically multipotent, producing all major retinal neurons regardless of the neuronal cell type ablated or the stimulus that triggers the regenerative event. Moreover, these data suggest that this process stimulates the generation of excessive amounts of neurons. This is an important consideration. If excess neurons are generated, it would become important to investigate whether or not they persist and impact retinal structure or function. Nonetheless, the implications of these previous studies remains unclear because growth factor and cytokine treatment of uninjured retinas exposes the retina to a unique stimulus relative to a normal retinal injury, potentially altering MG progenitor multipotency.

Studies of retina development and regeneration suggest a number of possible mechanisms that may bias the fate of MG-derived progenitors. First, injury-responsive MG phagocytose dying retinal neurons, and this may provide a substance that influences progenitor fate^25^. Second, progenitor fate may be controlled by diffusible signals emanating from dying or surviving cells. Indeed, in the developing retinas of fish and mammals, retinal progenitors appear to receive feedback inhibition from retinal neurons which then limit the generation of those neurons^26^,^27^,^28^. Furthermore, in the larval frog retina, the ablation of specific neurons biases progenitors towards regenerating those neurons over other cell types^29^,^30^. Finally, in the adult fish retina, surviving cells seem to enhance retina regeneration, though the mechanism stimulating this enhancement remains unknown^31^.

Here we investigated the fate of MG-derived progenitors using three different injury models that ablated unique populations of retinal neurons. Our studies suggest that following retinal injury, MG-derived progenitors are intrinsically programmed to regenerate all major retinal cell types regardless of which cells are initially damaged. Thus, new neurons are seeded into undamaged retinal layers. Interestingly, we find that progenitors proliferate more when they migrate to cell layers where retinal neurons have been ablated compared to when they migrate to these layers without neuronal ablation, perhaps suggesting a mechanism of feedback inhibition.

## Results

### Unique cell death signatures stimulate MG proliferation in the INL

We took advantage of three different neuronal ablation paradigms to investigate if the multipotency of MG-derived progenitors was impacted by the population of neurons ablated. Previous studies have documented that a mechanical injury with a needle poke damages neurons in all retinal layers and that photoablation with intense UV light selectively damages photoreceptors in the outer nuclear layer (ONL)^3^,^32^. To selectively damage neurons in the inner nuclear layer (INL) and ganglion cell layer (GCL), but spare photoreceptors, we took advantage of NMDA-mediated neurotoxicity that was reported to damage these neurons in the goldfish retina^33^.

**Figure 1:**
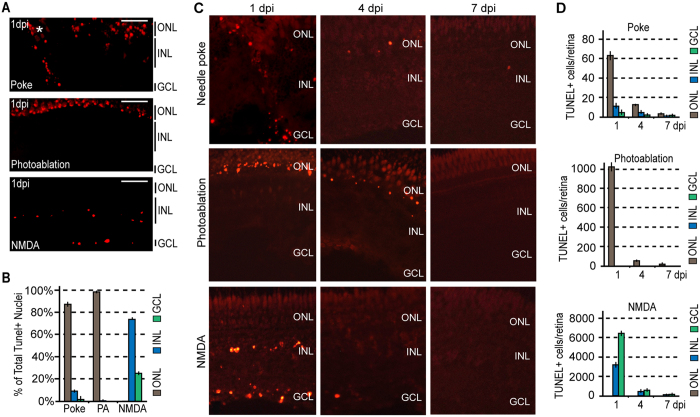
Injury models generate unique cell death signatures. (**A**) Representative images of retinal sections showing TUNEL detection (red) of apoptotic neuronal cell death 1 day following needle poke, UV light photoablation (PA), and NMDA neurotoxic injuries. (**B**) Relative localization of TUNEL+ nuclei by nuclear layer in the various injury models at 1 dpi. (**C**) Representative images of retinal sections showing TUNEL detection of apoptotic neuronal cell death at 1, 4 and 7 days following needle poke (single injury/retina), UV light photoablation, and NMDA neurotoxic injuries. (**D**) Quantification of TUNEL+ cells by nuclear layer in the various injury models at 1, 4 and 7 dpi. Data represents means ± s.d. (n = 4). Scale bar is equal to 50 μm. ONL, outer nuclear layer; INL, inner nuclear layer; GCL, ganglion cell layer; PA, photoablation; dpi, days post injury.

We used TUNEL assays to quantify retinal layer-specific cell death with each of these paradigms at 1 day post injury (dpi) (Fig. 1A,B). Although TUNEL assay is generally associated with apoptosis, it has been reported to also detect necrosis and autolytic cell death^34^. However, the initiation of DNA damage may vary, and for this reason some dying cells may go undetected at any particular time point of analysis. Importantly, a needle poke injury stimulated cell death in all retinal layers, photoablation restricted cell death to the ONL, and NMDA neurotoxicity restricted cell death to the INL and GCL (Fig. 1). The relatively low level of cell death noted in the INL and GCL after a needle poke injury is likely an underestimate as many of these neurons are mechanically displaced into the vitreous as the needle is inserted through the retina and therefore, could not be included in cell death counts because their layer of origin could not be determined. Our analysis of cell death at 1, 4 and 7 dpi indicated that the majority of cell death occurs at 1 dpi in all injury paradigms with little to no detectable cell death occurring at 4 and 7 dpi (Fig. 1C,D).

**Figure 2:**
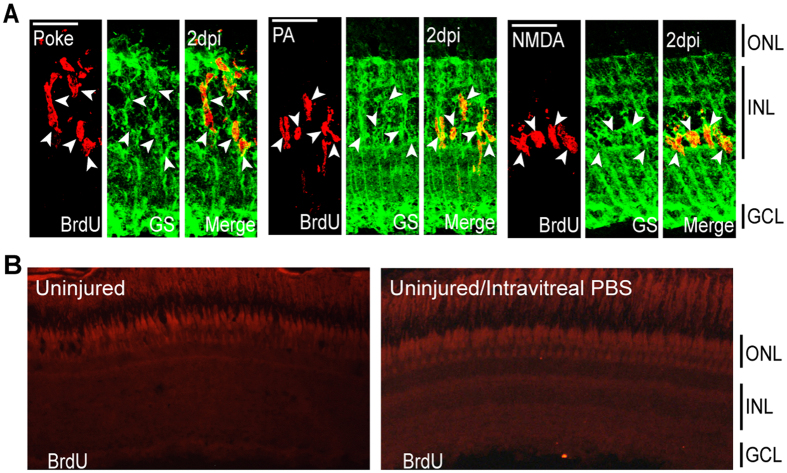
Injury models stimulate MG proliferation by 2 dpi in the INL. (**A**) Representative confocal images of retinal sections immunostained for BrdU and GS at 2 dpi following needle poke, PA, and NMDA injuries. Fish were given an injection of BrdU intraperitoneally 3 hours before harvest. Scale bar is equal to 50 μm. (**B**) PBS was injected into the vitreous of eyes whose retinas were uninjured. Fish were then given an injection of BrdU intraperitoneally 3 hours before harvest at 2 days post PBS injection. Shown are representative images of BrdU immunofluorescence in retinal sections. Similar results were obtained when BrdU was injected at 4 days post PBS injection. ONL, outer nuclear layer; INL, inner nuclear layer; GCL, ganglion cell layer; PA, photoablation; GS, glutamine synthetase; dpi, days post injury.

To investigate the early response of MG to the various injury paradigms, fish received an intraperitoneal (IP) injection of BrdU at 2 dpi when MG are just beginning to proliferate^3^,^7^. These fish were sacrificed 3 hours later, and retinal sections were processed for BrdU and glutamine synthetase (GS) immunofluorescence to investigate injury-dependent MG proliferation (Fig. 2A). This analysis showed that all injury paradigms selectively stimulate MG proliferation in the INL. No MG proliferation was detected in control uninjured retinas (Fig. 2B). These results are consistent with previous studies showing that MG respond to retinal injury by dividing and generating a proliferating population of retinal progenitors^3^,^18^,^19^,^20^,^32^.

### Regardless of the injury paradigm, MG-derived progenitors migrate to all three retinal nuclear layers and generate cells that persist for long periods of time

We next investigated if the injury paradigms uniquely impacted the behavior of MG-derived progenitors at later stages of the regenerative response. For this analysis fish received an IP injection of BrdU at 2 dpi, which labeled the MG-derived progenitors. Fish were then sacrificed at 2 (3 hours post BrdU injection), 4, 7, 14, or 30 dpi, and the ratio of BrdU+ cells in each retinal nuclear layer was determined to investigate if progenitor proliferation or lamination was impacted by the injury paradigm.

**Figure 3:**
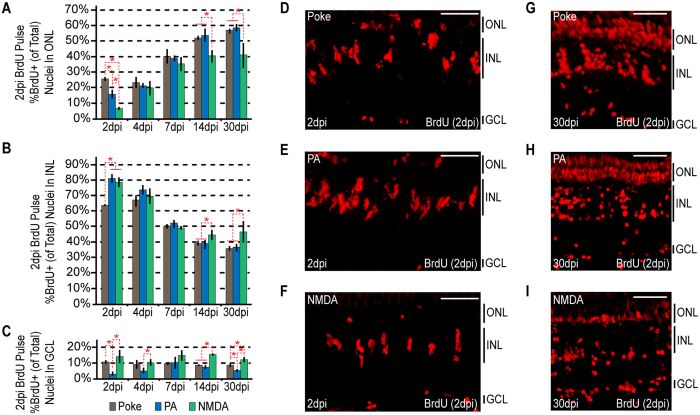
Injury paradigms stimulate regenerative response signatures that exhibit both commonality and uniqueness. Fish were given an intraperitoneal injection of BrdU at 2 dpi and then sacrificed at 2 dpi (3 hours post BrdU injection), 4, 7, 14 and 30 dpi. (**A–C**) BrdU+ nuclei were counted and the percentage of BrdU+ nuclei residing in the (**A**) ONL, (**B**) INL and (**C**) GCL was determined for individual samples for each injury model. Data represents means ± s.d. (n ≥ 3). *P < 0.04549. (**D**–**I**) Representative images of retinal sections analyzed in (**A**–**C**) that were immunostained for BrdU at (**D**–**F**) 2 dpi or (**G**–**I**) 30 dpi following (**D**,**G**) needle poke, (**E**,**H**) PA or (**F**–**I**) NMDA injury. Scale bar is equal to 50 μm. Abbreviations are as in Fig. 1.

We found that the 3 different injury paradigms exhibited differences in the relative localization of BrdU+ cells at 2 dpi: a needle poke injury and photoablation resulted in more BrdU+ cells in the ONL than did NMDA neurotoxicity (Fig. 3A); photoablation and NMDA neurotoxicity resulted in more BrdU+ cells in the INL than did a needle poke injury (Fig. 3B); and a needle poke injury and NMDA neurotoxicity resulted in more BrdU+ cells in the GCL than did photoablation (Fig. 3C). At 2 dpi, the number of retinal progenitors is relatively low, so these ratio differences might be attributed to slight timing variations between the injury models or to other cell populations, such as microglia and rod progenitors, that under certain circumstances have been noted to proliferate following retinal injury^14^,^35^,^36^. Consistent with these ideas, the differences seen in the localization of BrdU+ cells between the injury paradigms almost completely disappear around 4–7 dpi (Fig. 3A–C) by which time the BrdU+ MG-derived progenitors have achieved maximal rates of proliferation^3^,^7^.

Interestingly, at later times post injury (14–30 dpi) we observed a correlation between the ratio of BrdU+ cells occupying a particular retinal nuclear layer and the ablated cell type (Fig. 3A–C). For example, we noted a higher ratio of BrdU+ cells in the ONL of retinas where photoreceptors were preferentially ablated using either a needle poke or photoablation model of injury compared to retinas where photoreceptors were spared as in the NMDA neurotoxin model of injury (Fig. 3A). Likewise, we observed a higher ratio of BrdU+ cells in the INL and GCL when cells in these layers were selectively ablated with NMDA compared to when photoreceptors were preferentially ablated with UV light or a needle poke injury (Fig. 3B,C). Thus, there does seem to be a correlation between the ratio of BrdU+ cells residing in a particular retinal layer and the ablated cell type by 14–30 dpi.

Finally, we noted that in all injury paradigms, BrdU+ cells migrate to and occupy all retinal nuclear layers (Fig. 3A–I). Thus, specific ablation of photoreceptors does not preclude MG-derived progenitors from migrating and taking up residence in the INL or GCL, nor does specific ablation of INL or GCL neurons preclude MG-derived progenitors from migrating and taking up residence in the ONL. Thus, MG-derived progenitors are intrinsically multipotent and generate new neurons that occupy undamaged retinal layers.

### MG-derived progenitors proliferating later in the regenerative response demonstrate greater injury paradigm-specific biases than those proliferating earlier

**Figure 4:**
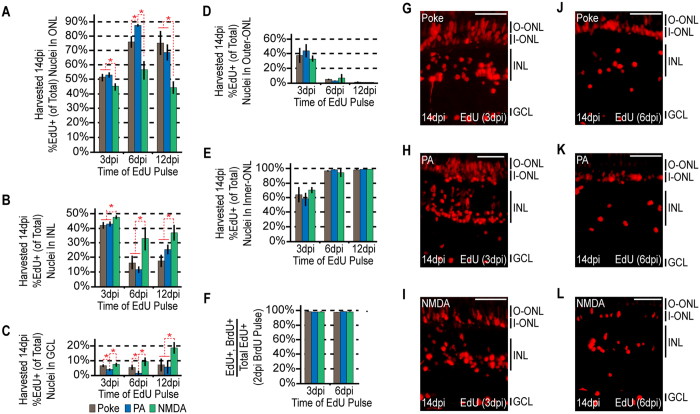
The ultimate localization of MG-derived progenitors becomes progressively more biased at later rounds of proliferation. Fish were given an intraperitoneal injection of BrdU at 2 dpi, followed by an injection of EdU at 3, 6, or 12 dpi. Each sample was harvested at 14dpi. (**A**–**C**) EdU+ nuclei were counted at the times indicated and the percentage of EdU+ nuclei residing in the (**A**) ONL, (**B**) INL and (**C**) GCL was determined for each injury model. Data represents means ± s.d. (n ≥ 3). *P < 0.02941. (**D**,**E**) The percentage of ONL EdU+ nuclei residing in (**D**) the upper region or (**E**) the lower region was determined for each injury model. (**F**) Co-staining samples for BrdU and EdU demonstrates that the cells proliferating at later times are a subpopulation of those proliferating at earlier times. (**G**–**L**) Representative images of retinal sections analyzed in (**A–F**) that were stained for EdU at (**G**–**I**) 3 dpi or (**J**–**L**) 6 dpi following (**G**,**J**) needle poke, (**H**,**K**) PA or (**I–L**) NMDA injury. Scale bar is equal to 50 μm. Abbreviations are as in Fig. 1.

The above data indicate that although MG-derived progenitors contribute cells to all nuclear layers regardless of the injury paradigm, the ratio of BrdU+ cells localized to each layer is ultimately biased by which neurons were ablated. It is possible that disparities in progenitor cell migration and/or dissimilar levels of progenitor proliferation after migration generate these differences. In an attempt to uncover how these differences between injury paradigms arise, injured fish were given an IP injection of EdU at 3, 6 or 12 dpi and sacrificed at 14 dpi. Retinal sections were prepared and stained for EdU incorporation. We found that progenitors labelled with EdU at 3 dpi and harvested at 14 dpi showed comparable injury-paradigm dependent biases to those labeled with BrdU at 2 dpi and harvested at 14 dpi (Figs 3A–C and 4A–C). However, these biases increased with EdU pulses at 6 or 12 dpi (Fig. 4A–C,G–L). These results suggest that the ablated cell types impact MG-derived progenitor proliferation less at early times than at later times in the regenerative response. Thus, it is possible that the retinal progenitors receive feedback inhibition from surviving neurons that suppresses their proliferation.

During the course of these experiments we noted that the occupation of EdU+ nuclei in the outer and inner portion of the ONL at 14 dpi varied depending on when EdU was delivered post injury (Fig. 4D,E,G–L). For progenitors occupying the outer-most portion of the ONL, EdU labelling suggested they proliferated at 3 dpi, but largely stopped proliferating by 6 dpi (Fig. 4D). In contrast, progenitors occupying the inner-most portion of the ONL could be labelled with EdU+ at all the time points examined (3, 6 and 12 dpi, Fig. 4E). Although the reason for these differences is not known, it may suggest that proliferating progenitors migrating into the outer portion of the ONL encounter a transient injury-dependent permissive environment for proliferation/migration that becomes more restrictive as time goes on.

Although our above data suggested that the retinal environment affects progenitor proliferation (feedback inhibition by surviving neurons) and migration (differences in occupancy of the outer and inner portions of the ONL), it is possible that this actually reflects 2 different proliferating progenitor populations that were labelled at different times of EdU application. To test this, we injured the retina and then gave fish an IP injection of BrdU at 2 dpi when MG are just beginning to proliferate^3^,^7^. This was followed by an IP injection of EdU at 3 or 6 dpi, and fish were then sacrificed at 14 dpi. BrdU and EdU assays indicated that essentially 100% of the EdU+ cells proliferating at 3 and 6 dpi began proliferating at 2 dpi and this was true for all our injury paradigms (Fig. 4F). Thus, no new MG are entering the cell cycle at the later time points.

### Enhanced progenitor proliferation in retinal layers with ablated cells

**Figure 5:**
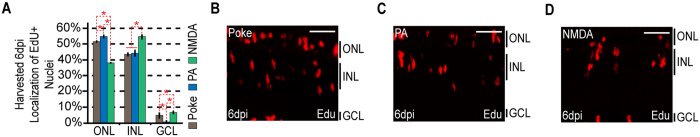
The localization of proliferating MG-derived progenitors by 6 dpi demonstrate injury-specific biases. Fish were given an injection of EdU 2 hours before harvesting at 6 dpi. (**A**) EdU+ cells were counted and the percentage of EdU+ nuclei residing in the ONL, INL, and GCL was determined for each injury model. Data represents means ± s.d. (n ≥ 3). *P < 0.02764. (**B–D**) Representative images of retinal sections analyzed in (**A**) that were stained for EdU following (**B**) needle poke, (**C**) PA or (**D**) NMDA injury. Scale bar is equal to 50 μm. Abbreviations are as in Fig. 1.

The above data suggested that MG-derived progenitors/neurons may receive feedback inhibition from surviving neurons to limit their proliferation or survival at late stages of regeneration. To directly test if progenitor proliferation was affected, injured fish were given an IP injection of EdU at 6 dpi and sacrificed 2 hours later. Retinal sections were prepared and assayed for EdU incorporation. Quantification of EdU+ cells in each retinal layer for each type of injury revealed that progenitor proliferation is enhanced in the ONL when photoreceptors are ablated (needle poke and photoablation) compared to when progenitors occupy an intact ONL (NMDA) (Fig. 5A–D). Similarly, progenitor proliferation is enhanced in the INL and GCL when resident neurons are ablated (needle poke and NMDA) compared to when progenitors occupy an intact INL and GCL (photoablation) (Fig. 5A–D). These data suggest that progenitors receive feedback inhibition from surviving retinal neurons that limits their proliferation and that this inhibition is relieved by cell ablation.

### Regardless of the injury paradigm, MG-derived progenitors are multipotent and can generate excess neurons that seed undamaged retinal layers

Previous studies have established that MG-derived progenitors migrate to retinal nuclear layers that are appropriate for the cell types they regenerate^3^,^7^,^22^,^32^,^37^. Thus, photoreceptors are regenerated by progenitors laminating to the ONL; horizontal, bipolar and amacrine neurons are regenerated by progenitors laminating to the INL; and ganglion cells are regenerated by progenitors laminating to the GCL. Our data indicate that regardless of the injury paradigm, MG-derived progenitors migrate and take up residence in all retinal layers regardless of whether that layer was damaged. This is most evident for the photoablation model where progenitors are found to occupy INL and GCL layers, and also for NMDA damaged retinas where progenitors are found to occupy the ONL. Furthermore, our data suggests new neurons persist in these layers for at least one month.

**Figure 6:**
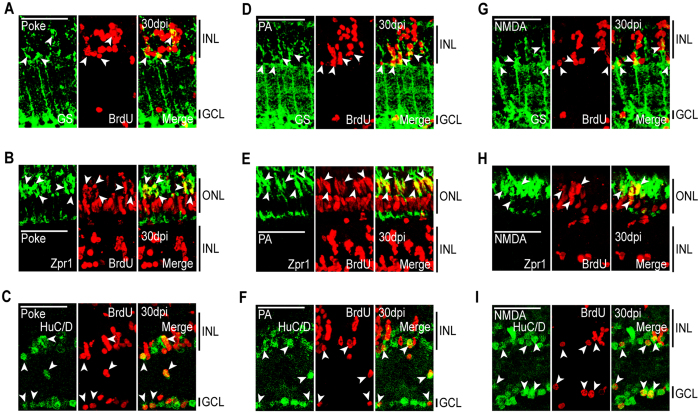
MG-derived progenitors are multipotent and generate unneeded neurons. Fish were given an intraperitoneal injection of BrdU at 2 dpi and sacrificed at 30 dpi. Immunostaining for BrdU and retinal cell type-specific markers was then performed against: GS, MG; Zpr1, red/green cones; HuC/D, amacrine and ganglion cells. Representative immunofluorescence images of retinal sections following (**A**–**C**) needle poke, (**D**–**F**) PA, or (**G–I**) NMDA injury. Retinal sections were stained with antibodies detecting BrdU and (**A,D,G**) GS, (**B**,**E**,**H**) Zpr1, or (**C**,**F**,**I**) HuC/D. Scale bar is equal to 50 μm. Abbreviations are as in Fig. 2.

Although previous studies have demonstrated that progenitors migrating to retinal layers differentiate into layer appropriate neurons, we confirmed this is also true when they migrated to undamaged retinal layers. For this analysis, injured fish received an IP injection of BrdU at 2 dpi and were sacrificed at 30 dpi. Retinal sections were then analyzed for BrdU and retinal cell type-specific immunofluorescence. In retinas with a needle poke injury that damaged all retinal layers, we detected regenerated (BrdU+) cells that co-expressed the MG marker, GS; the red/green cone marker, Zpr1; and the amacrine and ganglion cell marker, HuC/D, in the INL and GCL (Fig. 6A–C). In retinas where damage was restricted to photoreceptors (PA model) we not only identified regenerated photoreceptors (BrdU+/Zpr1+) in the ONL, but also regenerated amacrine and ganglion cells (BrdU+/HuC/D+) in the INL and GCL (Fig. 6D–F). In retinas with damage restricted to the INL and GCL (NMDA model), we detected regenerated amacrine and ganglion (BrdU+/HuC/D+) cells in the INL and GCL, respectively, along with regenerated photoreceptors (BrdU+/Zpr1+) in the ONL (Fig. 6G–I). As expected, in all instances we identify BrdU+/GS+ MG in the INL whose cell division was responsible for progenitor formation (Fig. 6). Thus, MG-derived progenitors are not restricted to regenerating only ablated cell types, but rather seem to be programmed to produce all major retinal cell types that seed all retinal layers with new neurons regardless of the injury paradigm.

## Discussion

In the damaged fish retina, a proliferating population of MG-derived progenitors in the INL are responsible for retinal repair^3^,^5^. These progenitors have been observed to accumulate in regions of cell damage and regenerate lost neurons^3^,^32^,^38^. Indeed, MG-derived progenitors can be found to migrate to all retinal nuclear layers after a needle poke or retinal extraction injury that damages all retinal cell types^3^,^19^,^39^, while more restrictive injuries that specifically ablate photoreceptors or inner retinal neurons have been reported to result in more selective migration of progenitors to these injured areas^22^,^23^,^24^. This latter observation suggests that MG-derived progenitors may be able to sense cell loss and bias regeneration towards that particular cell type. Indeed previous studies have suggested that selective cone ablation results in preferential regeneration of cones, while selective ablation of inner retinal neurons results in their regeneration without new neurons being added to the ONL^22^,^23^. However, these studies were imprecise in their analysis because they relied on nuclear counts for quantifying regeneration and did not assay for all retinal cell types. We had previously shown that even in an undamaged retina, intravitreal injection of growth factors and cytokines can stimulate MG to divide and generate progenitors that seed all retinal layers with new neurons^12^,^14^, suggesting these progenitors are intrinsically multipotent and that the influence of surviving or dying neurons on this multipotency is relatively small. However, because growth factors and cytokines may have unforeseen consequences on MG-derived progenitors that is not reflective of a retinal injury, it remained unclear if MG-derived progenitors in the injured retina exhibited restricted fates that were biased towards ablated cell types.

To investigate the multipotential character of progenitors in the injured retina, we used three different injury models that ablate different or overlapping cell populations. We used BrdU+ nuclear counts to quantify changes in cell proliferation. Because stereological methods were not employed these counts may be biased towards higher numbers. However, because all counts were done the same way, this bias should be uniform across samples and the reported differences between samples unaffected by a lack of stereology.

Our studies suggest that MG-derived progenitors are intrinsically multipotent and seed all retinal layers with new neurons regardless of whether that layer was damaged. Furthermore, our studies indicate that MG-derived progenitors receive feedback inhibition from retinal neurons and that this inhibition reduced proliferation in uninjured retinal layers, which may explain previously noted biases in cell type-specific regeneration.

MG proliferation, migration and differentiation are key components of successful regeneration and have been well studied. Therefore, we focused on these aspects of regeneration: from 2 dpi, when MG just begin to enter the cell cycle, through 30 dpi, when MG-derived progenitors have migrated to their final positions and taken up residence in their appropriate nuclear layers as differentiated cells. Importantly, our data suggest that regardless of what cell types are ablated, MG initially respond in a similar fashion by entering the cell cycle in the INL and generating progenitors that migrate to and populate all retinal nuclear layers. This suggests MG do not sense the type of cell that was injured, but more likely are responding to an injury signal common to all cell ablations. This lends little support to the idea that phagocytosis of debris from dying cells influences MG’s ability to regenerate specific cell types^25^. However, at later times post injury (14–30 dpi) when progenitors have migrated to their final destination, we found that their laminar distribution is biased toward layers where neurons were ablated. This suggested to us that the later divisions of MG-derived progenitors may be regulated by neurons in the progenitor’s environment. Indeed, we found that cell ablation had only small effects on progenitor proliferation at 3 dpi when proliferation is initiated, but becomes much more dramatic at 6–12 dpi when progenitors are closer to their final divisions.

Remarkable is the observation that all injury paradigms result in progenitors migrating to and differentiating in all retinal layers regardless of whether cells were ablated. This suggests an inefficient regeneration process that generates excess and ectopic cell types. Indeed, our BrdU-based lineage tracing showed that HuC/D+ neurons are generated in the INL and GCL of photoreceptor ablated retinas, and that Zpr1+ photoreceptors are generated in the NMDA-damaged retina that ablates cells selectively from the INL and GCL. Our data, along with others^39^, also suggests excess/ectopic neurons are generated in the mechanically damaged retina. It is unlikely these cells are simply progenitors migrating through these layers since they are detected at 1 month post injury and express neuron markers that are appropriate for the cell types that reside in these layers. It is also unlikely that these are nuclei exhibiting interkinetic nuclear transfer that is observed in dividing cells since there are essentially no proliferating cells in these regions of the retina at 1 month post injury^3^,^40^. Although the consequence of generating excess neurons is not known, it would be important to determine if: 1) they integrate into existing circuits or make new ones; 2) contribute to retinal growth; and 3) impact retinal health by replacing older neurons with younger ones.

## Materials and Methods

### Animals

Zebrafish were kept at 26–28 °C on a 14/10 hr light/dark cycle. All experiments were performed on Wt or *1016 tuba1a:gfp* transgenic fish^3^,^41^. Fish were harvested by treatment with a lethal dose of tricaine methane sulfonate (Sigma). Only adult fish (≥3 months of age) were used in this study. Results from experiments performed using *1016 tuba1a:gfp* were verified using Wt fish. No significant differences were noted. All experimental protocols were approved by University of Michigan’s Committee on Use and Care of Animals. Methods were carried out in accordance with the approved guidelines.

### Retinal Injury

Mechanical lesions have been described previously^3^,^41^. Briefly, fish were anesthetized in 0.02% tricaine methane sulfonate (Sigma) and under microscopic visualization eyes were gently rotated in their sockets and stabbed through the sclera with a 30 gauge needle to the depth of the bevel.

Light lesions (photoablation, PA) were carried out as has been described previously^32^. Briefly, fish were exposed to intense UV light for 30 minutes. An EXFO X-Cite 120W metal halide lamp (EXFO Photonic Solutions, Mississauga, Ontario, Canada) served as the light source.

Chemical lesions were carried out by intravitreal injections of 0.75 μL of 100 mM NMDA (Sigma). Intravitreal injections were carried out as described previously^14^. Briefly, fish were anesthetized in 0.02% tricaine methane sulfonate and under microscopic visualization a small incision was produced in the cornea with either a double-edge sapphire blade (World Precision Instruments) or a 30G needle. If a 30G needle was used to make the incision, small surgical scissors were used to slightly increase the size of the incision. A Hamilton syringe equipped with a blunt 30G needle was then used to deliver molecules behind the lens.

Controls included uninjured retinas and PBS injected into the vitreous of uninjured retinas.

### BrdU and EdU Injections

At the times specified, fish were given a 20 μL intraperitoneal injection of 20 mM BrdU or 10 mM EdU dissolved in ddH_2_0.

### Tissue Preparation

The eyes from adult zebrafish were enucleated, followed by the removal of the lens and immersion into fresh 4% paraformaldehyde in 0.1 M phosphate buffer, pH 7.4, for 2–3 hours at room temperature. After fixation, tissues were cryoprotected in phosphate-buffered 20% sucrose before embedding with Tissue-Tek O.C.T. compound (Sakura, Finetek). Embedded samples were sectioned to 12 microns on a CM3050S cryostat (Leica). Sections were collected on Superfrost/Plus slides (Fisher Scientific), dried and stored at −20 °C.

### Immunofluorescence

Immunofluorescence was performed as described previously^19^ using the following primary antibodies: rat anti-BrdU (dividing cell marker, 1:500, Thermo Scientific), mouse anti-Zpr1 (red/green cones, 1:250, ThermoFisher), mouse anti-GS (Müller glia, 1:500, Millipore), and mouse anti-HuC/D (1:300, amacrine and ganglion cells, ThermoFisher). The following secondary antibodies were used: Alexa Flour 488 donkey anti-mouse IgG (1:1000, ThermoFisher), Cy5 donkey anti-mouse (1:1000, ThermoFisher), Alexa Four 488 goat anti-rat IgG (1:1000, ThermoFisher), and Cy3 donkey anti-rat (1:1000, Jackson Immunoresearch). Antigen retrieval for BrdU staining was performed by either boiling the sections in 10 mM sodium citrate for 20 min and cooling for another 20 min or treating the sections with 2 N HCl at 37 °C for 25 min, followed by a rinse with 0.1 M sodium borate solution (pH 8.5) for 5 min.

### EdU Staining

Permeabilization of tissue in preparation for EdU detection was carried out by incubation of the slides with 0.5% Triton X-100 in PBS. Detection was then carried out using the reagents of the Click-iT EdU Imaging Kit (Invitrogen) according to the protocol outlined by the manufacturer.

### TUNEL Assay

TUNEL assays were performed on slides treated for 22 min with Proteinase K (Roche) at 37 °C (10 ug/ml Proteinase K, 10 mM Tris-HCl pH 7.5). Staining was carried out using the *In situ* Cell Death Detection Kit, TMR Red (Roche).

### BrdU, EdU, TUNEL Counts

Counts of BrdU+, EdU+, or TUNEL+ nuclei were performed by either counting the total number of stained nuclei across ≥5 retinal sections of an individual sample or by counting the total number of stained nuclei across ≥5 images of a sample’s injury site. The presence of an injury response was used in the selection of sections used for counting. We avoided the retinal periphery where some stem cells reside and are responsible for retinal expansion, but not regeneration^42^. Counts were done blindly and independently by two individuals. The average number of positive nuclei counted across each sample was as follows: Fig. 1, 1 dpi TUNEL counts: 407 nuclei. Figure 3, 2 dpi BrdU counts: 487 nuclei. Figure 3, 4 dpi BrdU counts: 1013 nuclei. Figure 3, 7 dpi BrdU counts: 1477 nuclei. Figure 3, 14 dpi BrdU counts: 2016 nuclei. Figure 3, 30d pi BrdU counts: 3748 nuclei. Figure 4, 3 dpi EdU counts: 1737 nuclei. Figure 4, 6 dpi EdU counts: 982 nuclei. Figure 4, 12 dpi EdU counts: 526 nuclei. Figure 5, 6 dpi EdU counts: 1449 nuclei. Statistics were run across ≥3 independent samples.

### Imaging and Statistics

Images were examined using a Zeiss Axiophot, Axio Observer Z.1 (for BrdU and TUNEL counts/analyses) or an Olympus FluoView FV1000 confocal imaging system (for BrdU, cell marker and BrdU, EdU co-localization analyses). Double labelling of cells was confirmed on single 0.5 μm thick optical sections. Images were captured using a digital camera adapted onto the microscope and were processed/annotated with Adobe Photoshop and Illustrator. Student T tests were performed to determine statistical differences between samples.

## Additional Information

**How to cite this article**: Powell, C. *et al.* Zebrafish Muller glia-derived progenitors are multipotent, exhibit proliferative biases and regenerate excess neurons. *Sci. Rep.* **6**, 24851; doi: 10.1038/srep24851 (2016).

## References

[b1] Hitchcock P. F. & Raymond P. A. The teleost retina as a model for developmental and regeneration biology. Zebrafish 1, 257–271 (2004). 18248236 10.1089/zeb.2004.1.257

[b2] Otteson D. C. & Hitchcock P. F. Stem cells in the teleost retina: persistent neurogenesis and injury-induced regeneration. Vision Res 43, 927–936 (2003). 12668062 10.1016/s0042-6989(02)00400-5

[b3] Fausett B. V. & Goldman D. A role for alpha1 tubulin-expressing Muller glia in regeneration of the injured zebrafish retina. J Neurosci 26, 6303–6313 (2006). 16763038 10.1523/JNEUROSCI.0332-06.2006 PMC6675181

[b4] Thummel R., Kassen S. C., Montgomery J. E., Enright J. M. & Hyde D. R. Inhibition of Muller glial cell division blocks regeneration of the light-damaged zebrafish retina. Dev Neurobiol 68, 392–408 (2008). 18161852 10.1002/dneu.20596 PMC3711086

[b5] Goldman D. Muller glial cell reprogramming and retina regeneration. Nature reviews. Neuroscience 15, 431–442 (2014). 24894585 10.1038/nrn3723 PMC4249724

[b6] Lamba D., Karl M. & Reh T. Neural regeneration and cell replacement: a view from the eye. Cell Stem Cell 2, 538–549 (2008). 18522847 10.1016/j.stem.2008.05.002 PMC2692223

[b7] Kassen S. C. *et al.* Time course analysis of gene expression during light-induced photoreceptor cell death and regeneration in albino zebrafish. Dev Neurobiol 67, 1009–1031 (2007). 17565703 10.1002/dneu.20362

[b8] Powell C., Grant A. R., Cornblath E. & Goldman D. Analysis of DNA methylation reveals a partial reprogramming of the Muller glia genome during retina regeneration. Proc Natl Acad Sci USA 110, 19814–19819 (2013). 24248357 10.1073/pnas.1312009110 PMC3856824

[b9] Ramachandran R., Fausett B. V. & Goldman D. Ascl1a regulates Muller glia dedifferentiation and retinal regeneration through a Lin-28-dependent, let-7 microRNA signalling pathway. Nat Cell Biol 12, 1101–1107 (2010a). 20935637 10.1038/ncb2115 PMC2972404

[b10] Qin Z., Barthel L. K. & Raymond P. A. Genetic evidence for shared mechanisms of epimorphic regeneration in zebrafish. Proc Natl Acad Sci USA 106, 9310–9315 (2009). 19474300 10.1073/pnas.0811186106 PMC2695073

[b11] Ramachandran R., Zhao X. F. & Goldman D. Ascl1a/Dkk/{beta}-catenin signaling pathway is necessary and glycogen synthase kinase-3{beta} inhibition is sufficient for zebrafish retina regeneration. Proc Natl Acad Sci USA 108, 15858–15863 (2011). 21911394 10.1073/pnas.1107220108 PMC3179085

[b12] Wan J., Ramachandran R. & Goldman D. HB-EGF is necessary and sufficient for Müller glia dedifferentiation and retina regeneration. Dev Cell 22, 334–347 (2012). 22340497 10.1016/j.devcel.2011.11.020 PMC3285435

[b13] Zhao X. F. *et al.* Leptin and IL-6 Family Cytokines Synergize to Stimulate Muller Glia Reprogramming and Retina Regeneration. Cell Reports 9, 272–284 (2014). 25263554 10.1016/j.celrep.2014.08.047 PMC4194149

[b14] Wan J., Zhao X. F., Vojtek A. & Goldman D. Retinal Injury, Growth Factors, and Cytokines Converge on beta-Catenin and pStat3 Signaling to Stimulate Retina Regeneration. Cell Reports 9, 285–297 (2014). 25263555 10.1016/j.celrep.2014.08.048 PMC4194164

[b15] Kassen S. C. *et al.* CNTF induces photoreceptor neuroprotection and Muller glial cell proliferation through two different signaling pathways in the adult zebrafish retina. Exp Eye Res 88, 1051–1064 (2009). 19450453 10.1016/j.exer.2009.01.007

[b16] Conner C., Ackerman K. M., Lahne M., Hobgood J. S. & Hyde D. R. Repressing notch signaling and expressing TNFalpha are sufficient to mimic retinal regeneration by inducing Muller glial proliferation to generate committed progenitor cells. J Neurocsci 34, 14403–14419 (2014). 10.1523/JNEUROSCI.0498-14.2014 PMC4205560 25339752

[b17] Nelson C. M. *et al.* Stat3 defines three populations of Muller glia and is required for initiating maximal muller glia proliferation in the regenerating zebrafish retina. J Comp Neurol 520, 4294–4311 (2012). 22886421 10.1002/cne.23213 PMC3478445

[b18] Nagashima M., Barthel L. K. & Raymond P. A. A self-renewing division of zebrafish Muller glial cells generates neuronal progenitors that require N-cadherin to regenerate retinal neurons. Development 140, 4510–4521 (2013). 24154521 10.1242/dev.090738 PMC3817940

[b19] Ramachandran R., Reifler A., Parent J. M & Goldman D. Conditional gene expression and lineage tracing of tuba1a expressing cells during zebrafish development and retina regeneration. J Comp Neurol 518, 4196–4212 (2010). 20878783 10.1002/cne.22448 PMC2948409

[b20] Ramachandran R., Zhao X. F. & Goldman D. Insm1a-mediated gene repression is essential for the formation and differentiation of Muller glia-derived progenitors in the injured retina. Nat Cell Biol 14, 1013–1023 (2012). 23000964 10.1038/ncb2586 PMC3463712

[b21] Thummel R. *et al.* Pax6a and Pax6b are required at different points in neuronal progenitor cell proliferation during zebrafish photoreceptor regeneration. Exp Eye Res 90, 572–582 (2010). 20152834 10.1016/j.exer.2010.02.001 PMC2856924

[b22] Fimbel S. M., Montgomery J. E., Burket C. T. & Hyde D. R. Regeneration of inner retinal neurons after intravitreal injection of ouabain in zebrafish. J Neurosci 27, 1712–1724 (2007). 17301179 10.1523/JNEUROSCI.5317-06.2007 PMC6673754

[b23] Fraser B., DuVal M. G., Wang H. & Allison W. T. Regeneration of cone photoreceptors when cell ablation is primarily restricted to a particular cone subtype. Plos One 8, e55410 (2013). 23383182 10.1371/journal.pone.0055410 PMC3559598

[b24] Hochmann S. *et al.* Fgf Signaling is Required for Photoreceptor Maintenance in the Adult Zebrafish Retina. Plos One 7, e30365 (2012). 22291943 10.1371/journal.pone.0030365 PMC3266925

[b25] Bailey T. J., Fossum S. L., Fimbel S. M., Montgomery J. E. & Hyde D. R. The inhibitor of phagocytosis, O-phospho-l-serine, suppresses Muller glia proliferation and cone cell regeneration in the light-damaged zebrafish retina. Exp Eye Res 91, 601–612 (2010). 20696157 10.1016/j.exer.2010.07.017 PMC2962682

[b26] Jusuf P. R. *et al.* Origin and determination of inhibitory cell lineages in the vertebrate retina. J Neurosci 31, 2549–2562 (2011). 21325522 10.1523/JNEUROSCI.4713-10.2011 PMC3083844

[b27] Poggi L., Vitorino M., Masai I. & Harris W. A. Influences on neural lineage and mode of division in the zebrafish retina *in vivo*. J Cell Biol 171, 991–999 (2005). 16365165 10.1083/jcb.200509098 PMC2171316

[b28] Belliveau M. J. & Cepko C. L. Extrinsic and intrinsic factors control the genesis of amacrine and cone cells in the rat retina. Development 126, 555–566 (1999). 9876184 10.1242/dev.126.3.555

[b29] Reh T. A. & Tully T. Regulation of tyrosine hydroxylase-containing amacrine cell number in larval frog retina. Dev Biol 114, 463–469 (1986). 2869994 10.1016/0012-1606(86)90210-1

[b30] Reh T. A. Cell-specific regulation of neuronal production in the larval frog retina. J Neurosci 7, 3317–3324 (1987). 3499488 10.1523/JNEUROSCI.07-10-03317.1987 PMC6569163

[b31] Sherpa T. *et al.* Retinal regeneration is facilitated by the presence of surviving neurons. Dev Neurobiol 74, 851–876 (2014). 24488694 10.1002/dneu.22167 PMC4106997

[b32] Bernardos R. L., Barthel L. K., Meyers J. R. & Raymond P. A. Late-stage neuronal progenitors in the retina are radial Muller glia that function as retinal stem cells. J Neurosci 27, 7028–7040 (2007). 17596452 10.1523/JNEUROSCI.1624-07.2007 PMC6672216

[b33] Tyan S. H., Sue T. Y., Hon Y. S., Gean P. W. & Chang Y. C. A novel NMDA receptor antagonist protects against N-methyl-D-aspartate- and glutamate-induced neurotoxicity in the goldfish retina. Eur J Pharmacol 321, 171–179 (1997). 9063685 10.1016/s0014-2999(96)00949-1

[b34] Grasl-Kraupp B. *et al.* *In situ* detection of fragmented DNA (TUNEL assay) fails to discriminate among apoptosis, necrosis, and autolytic cell death: a cautionary note. Hepatology 21, 1465–1468 (1995). 7737654 10.1002/hep.1840210534

[b35] Yurco P. & Cameron D. A. Responses of Muller glia to retinal injury in adult zebrafish. Vision Res 45, 991–1002 (2005). 15695184 10.1016/j.visres.2004.10.022

[b36] Fischer A. J., Zelinka C., Gallina D., Scott M. A. & Todd L. Reactive microglia and macrophage facilitate the formation of Muller glia-derived retinal progenitors. Glia 62, 1608–1628 (2014). 24916856 10.1002/glia.22703 PMC4140984

[b37] Ramachandran R., Reifler A., Wan J. & Goldman D. Application of Cre-loxP recombination for lineage tracing of adult zebrafish retinal stem cells. Methods Mol Biol 884, 129–140 (2012). 22688702 10.1007/978-1-61779-848-1_8

[b38] Vihtelic T. S. & Hyde D. R. Light-induced rod and cone cell death and regeneration in the adult albino zebrafish (Danio rerio) retina. J Neurobiol 44, 289–307 (2000). 10942883 10.1002/1097-4695(20000905)44:3<289::aid-neu1>3.0.co;2-h

[b39] Cameron D. A. Cellular proliferation and neurogenesis in the injured retina of adult zebrafish. Vis Neurosci 17, 789–797 (2000). 11153658 10.1017/s0952523800175121

[b40] Weber A. *et al.* Characterization of light lesion paradigms and optical coherence tomography as tools to study adult retina regeneration in zebrafish. Plos One 8, e80483 (2013). 24303018 10.1371/journal.pone.0080483 PMC3841302

[b41] Powell C., Elsaeidi F. & Goldman D. Injury-dependent Muller glia and ganglion cell reprogramming during tissue regeneration requires Apobec2a and Apobec2b. J Neurosci 32, 1096–1109 (2012). 22262907 10.1523/JNEUROSCI.5603-11.2012 PMC3306176

[b42] Raymond P. A., Barthel L. K., Bernardos R. L. & Perkowski J. J. Molecular characterization of retinal stem cells and their niches in adult zebrafish. BMC Dev Biol 6, 36 (2006). 16872490 10.1186/1471-213X-6-36 PMC1564002

